# Prevalence and Social Contextual Factors of Smokeless Tobacco Use: Insights from Schools of Delhi, India

**DOI:** 10.31557/APJCP.2021.22.8.2351

**Published:** 2021-08

**Authors:** Akash Deep Sharma, Suneela Garg, Meghachandra M Singh, Chetana Prakash Deshmukh, Pragya Sharma, Amod L Borle

**Affiliations:** *Department of Community Medicine, Maulana Azad Medical College, Delhi, India. *

**Keywords:** Smokeless tobacco, adolescents, school

## Abstract

**Background:**

It was observed that adult users start tobacco use in childhood or adolescence. The influence of digital and print media, social acceptance among peers, and in order to mimic role models from films attracts youth towards tobacco. Hence this study was conducted to assess the prevalence of smokeless tobacco (SLT) use among school-going adolescents with the assessment of the influencing factors such as exposure at school, home, and public places along with the role of various media in SLT use by adolescents.

**Methods:**

Cross sectional study was conducted with 860 students of class 9^th^ -12^th^ enrolled in schools. Purposive sampling of three schools was done from the study area to reach desired sample size. All the students of class 9^th^ - 12^th^ within selected schools were given chance to participate in the study. Data collection was done using pretested modified Global Youth Tobacco Survey (GYTS) questionnaire.

**Results:**

Consumption of smokeless tobacco (SLT) was observed in 79 (11%) of 714 subjects. Significantly higher proportion (18.4%) of study subjects consumed SLT who were exposed to tobacco at school premises and 19.1% of study subjects who were exposed to teacher using SLT in schools compared to non-exposed group. (p=0.016). It was observed that 8.1% of subjects without any exposure to tobacco at home and 9.8% of subjects without exposure to tobacco at outdoor public space consumed SLT. Consumption of SLT use was highest (16.7%) among subjects exposed to tobacco advertisement or promotion through social media (p=0.04).

**Conclusion:**

High prevalence of SLT was detected among adolescents in school. Factors such as exposure to tobacco at home, public places, school and school teacher using SLT, exposure of tobacco advertisement and promotion via different modes of media was found to be significantly associated with the use of SLT in the adolescents of school.

## Introduction

The World Health Organization (WHO) has defined ‘adolescents’ as individuals in the age group of 10 to 19 years (“WHO | Adolescent health in the South-East Asia Region,” n.d.).

Adolescents experience rapid physical, cognitive and psychosocial growth. This affects their emotional, social and psychological behaviour reflecting in their decision making and interaction with the world around them. It is a unique stage of human development and an important time for laying the foundations of good health (“WHO | Adolescent health,”n.d.).

It is estimated that 70% of premature deaths among adults are due to behavioral patterns that emerge in adolescence, including tobacco use, violence, and sexual behavior (“WHO |What about boys? A literature review on the health and development of adolescent boys,”2000).

It is observed that most of the adult users start tobacco use in childhood or adolescence (Chadda and Sengupta, 2002). It is also reported that youth initiating tobacco use in their adolescence will continue the consumption lifelong, with very low quitting rates (Thakur et al., 2014). 

Adolescents are the most vulnerable population to initiate tobacco use as it is a developmental phase where behavior of an individual is influenced by emotional and social factors. The influence of digital and print media, social acceptance among peers and in order to mimic role models from films or social media attracts youth towards tobacco consumption. It is observed that tobacco and alcohol use are seen as symbols of maturity and independence among the young people (Soni and Raut, 2013). 

Factors such as history of tobacco use by parents and elders at home , peer influence and pressure, the belief of association of tobacco use with masculinity and being “cool,” urge to experiment, underlying emotional and psychological problems have been reported to be reasons for pushing youth towards tobacco consumption (Oswal, 2015). Advertisements of different tobacco products are extremely common in all types of media including the print media, Television and road-side hoardings add onto tobacco consumption practices by the youth. Tobacco publicizing adequately target the youngsters by showing consumers as stylish, sporty and successful (Chadda and Sengupta, 2002; Vaidya et al., 1996).

Apart from home and social environment, adolescents spend most of the time in educational institutions. Teachers are in close contact with students in this course and students mimic their teachers. Therefore, teachers can be one of the important influencing factors to promote the tobacco consumption among the adolescents. Consumption of tobacco is common among school teachers, despite having awareness about the harmful effects of tobacco (Mishra et al., 2019).

According to the 2000 Global School Personnel Survey (GSPS) India, 78% of Bihar teachers used some form of tobacco. Later GSPS India (2006) showed nearly 40% of teachers in the eastern region of India, including Bihar used some form of tobacco, thus unknowingly promoting tobacco consumption (Nagler et al., 2015). Teachers represent key population for tobacco control efforts because they are role models for students, conveyor of tobacco prevention and influential members of society, capable of influencing both policies and social norms related to tobacco control (Nagler et al., 2015).

This study was conducted to assess prevalence of smokeless tobacco use among school going adolescents in senior secondary schools with the assessment of the influencing factors such as exposure at school, home and public places along with role of various media in smokeless tobacco use by adolescents.

## Materials and Methods

This was a cross sectional study conducted over a period of one year in the catchment areas within 10 km from rural health training center of Department of Community Medicine, Maulana Azad Medical College New Delhi which is located in Barwala village in North West district of Delhi in India. The study population comprised of all the students of class 9th -12th enrolled in schools present in the study area. Sample size was calculated using the formula ( n = Zα2 p q / L 2)taking the prevalence to be 12.5 % (“WHO | India (Ages 13-15) Global Youth Tobacco Survey (GYTS). Fact Sheet,” n.d.) at 95% confidence level with relative error of 20% and considering 10% non-respondents sample size was estimated to be 740.After obtaining clearance from institutional ethics committee and permission from School Health Scheme, Delhi, total 3schools were selected in the study area for the study. The principals and class teachers of the school were approached and explained the nature and purpose of the study after which the permission to conduct study in the school was obtained. On the first visit to school the study subjects were addressed for a period of 10-15 minutes in which the procedure and context of the study was explained and any queries regarding their involvement in the study were addressed at that time. The study subjects were given participant information sheet and consent forms for their parents to participate in the study. All those whose parents consented for the study their children were given assent forms on the day of study. After that they were given with a self- administered questionnaire in Hindi. Data collection from the study subjects of one school were done at one time only. Of the 860 study subjects approached for the study 714 gave consent from parents and assent to participate in the study. The data collected was entered in MS Excel and analyzed using SPSS version 25. Data were expressed in percentage. *χ*^2^ test was used for comparison of qualitative data between groups. ‘p’ value less than 0.05 was considered as significant.

## Results

*Characteristics of study subjects*

Table 1 show the characteristics of subjects who participated in the study. Consumption of smokeless tobacco (SLT) was observed in 79 (11%) of 714 study subjects. There were total 609 (85.3%) male participants out of which 62 (10.2%) were consuming SLT whereas among 105 (14.7%) female participants, 17 (16.2%) females consumed SLT. Maximum proportion of SLT consumers were from 12^th^ class (17.4%), living in joint families (12.0%) and having higher socioeconomic status (13.7%) The SLT consumption significantly increased with increase in class grade varying from 8.1 % in 9^th^ class to 17.4%in class 12^th^ (p=0.017) (Table 1).

A significantly higher proportion 51 (18.4%) of study subjects consumed SLT who were exposed to tobacco (smoked and non- smoked) at school premises and 17 (19.1%) of study subjects who were exposed to teacher using SLT in schools compared to non-exposed group.(p<0.0001, p=0.016) (Table 2).

It was observed that 23 (8.1%) of study subjects were not exposed to tobacco at home and 10 (9.8%) of study subjects were not exposed to tobacco at outdoor public spaces but still consumed SLT despite having nil exposure. Highest consumption of SLT was amongst 12 (20.7%) of study subjects who were exposed to tobacco at home for 5-6 days in a week(p=0.03) and 26(17.4 %) of study subjects who were exposed to tobacco at outdoor place for 7 days in a week compared to other categories of days of exposure per week (p=0.04) (Table 3).

Consumption of SLT use was significantly higher among 19 (16.7%) study subjects exposed through social media to tobacco advertisement or promotion than others exposed to television advertisements31 (10.6%), print media16 (9.5%) and exposed through radio 3(3.9%) (P= 0.04) (Table 4).

**Table 1:** Socio-Demographic Characteristicsof SLT Use among the Study Subjects (N=714)

Variables	no (%) N=714	SLT use no (%) (95% C.I)	χ2, df,‘p’ value
Class			
9^th^	223 (31.2)	18 (8.1) (4.8-12.5)	10.207, 3, 0.017
10^th^	215 (30.1)	18 (8.4) (5-12.9)	
11^th^	144 (20.2)	20 (13.9) (8.7-20.3)	
12^th^	132 (18.5)	23 (17.4) (11.38-24.99)	
Total	714	79 (11.0)(8.86-13.6)	
Gender			
Male	609 (85.3)	62 (10.2) (7.89-12.86)	3.287, 1, 0.07
Female	105 (14.7)	17 (16.2) (9.72-24.65)	
Religion			
Hindu	611 (85.6)	63 (10.3) (8.01-13)	2.546, 2, 0.280
Muslim	81 (11.3)	13 (16.0) (8.83-25.88)	
Others	22 (3.1)	3 (13.6) (2.91-34.91)	
Type of Family			
Nuclear	382 (53.7)	39 (10.2) (7.36-13.69)	0.610, 1, 0.435
Joint	332 (46.3)	40 (12.0) (8.75-16.04)	
Socioeconomic Status*			
High	234 (32.8)	32 (13.7) (9.55-18.75)	2.924, 2, 0.232
Upper Middle	170 (23.8)	19 (11.2) (6.86-16.90)	
Lower Middle	310 (43.4)	28 (9.0) (6.09-12.79)	

*, Modified B.G Prasad Scale -2019

**Table 2:** Exposure to Tobacco at School and SLT Use among Study Subjects (N= 714)

Exposure to	SLT use				
		Present no (%)	Absent no (%)	Total no (%)	χ2, df, ‘p’ value
Tobacco at school premise	Present	51 (18.4)	226 (81.6)	277 (100)	24.83. 1, <0.0001
	Absent	28 (6.4)	409 (93.6)	437 (100)	
	Total	79 (11)	635 (89)	714 (100)	
Teacher using SLT in school	Present	17 (19.1)	72 (80.9)	089 (100)	5.77, 1, 0.016
	Absent	62 (9.9)	563 (90.1)	625 (100)	
	Total	79 (11)	635 (89)	714 (100)	

**Table 3:** Exposure to Tobacco at Social Surroundings and SLT Use among Study Subjects (N=714)

Exposure to Tobacco	SLT use				
	Frequency (no of days/ week)	Present n (%)	Absent n (%)	Total N (%)	χ2, df, ‘p’ value
At home	0	23 (8.10)	260 (91.90)	283 (100)	8.37. 3, 0.03
	1-4	38 (11.50)	292 (88.50)	330 (100)	
	5-6	12 (20.70)	046 (79.30)	058 (100)	
	7	06 (13.95)	037 (86.05)	043 (100)	
	Total	79 (11)	635 (89)	714 (100)	
At outdoor public place	0	10 (9.80)	92 (90.20)	102 (100)	7.88, 3, 0.04
	1-4	27 (9.03)	272 (90.97)	299 (100)	
	5-6	16 (9.80)	148 (90.20)	164 (100)	
	7	26 (17.44)	123 (82.56)	149 (100)	
	Total	79 (11)	635 (89)	714 (100)	

**Table 4:** SLT Use in Relation to Exposure to Tobacco Advertisements via Different Mode of Media among Study Subjects

Forms of Media	SLT Use			
	Yes no (%)	No no (%)	Total no (%)	χ2, df,‘p’value
TV Advertisement	31 (10.6)	261 (89.4)	292 (100)	8.320, 3, 0.04
Print Media	16 (9.5)	153 (90.5)	169 (100)	
Social Media	19 (16.7)	95 (83.3)	114 (100)	
Radio Broadcast	3 (3.9)	74 (96.1)	77 (100)	
Total	69 (10.6)	583 (89.4)	652 (100)	

## Discussion

In the present study prevalence of SLT use was found to be 11 % which is similar to findings of the cross-sectional study conducted by Kumar et al., (2014) among 962 students of class 11^th^- 12^th^ in schools of Delhi in 2013, which reported the prevalence of ever tobacco chewers to be 12.5% . The prevalence in the present study was found to be slightly higher than reported by Mukherjee et al., (2012) among 276 students of 8^th^ -9^th ^class in schools of West Bengal in 2008 that reported 9.8% of the study participants had ever used SLT (Mukherjee et al., 2012). Study by Narain et al., (2011) among 4,786 students from 17 schools, reported a lower prevalence of 4.6% among the study subjects who were ‘ever tobacco chewer’ (Narain et al., 2011). This difference in the prevalence of smokeless tobacco use among these studies can be explained by the different study settings and different periods of time.

SLT consumption among our study subjects increased from (8.1% to 17 %) as the grades of their class increased from 9th to 12^th^. This finding was in accordance with the results of a cross-sectional study conducted by Chatterjee et al., (2016) among 10-18 years old school students of Mumbai which reported that the prevalence of smokeless tobacco consumption was 9% among students from 7^th^ class, 27% among students from 8th class and 64% among students from 9^th^ class (Chatterjee et al., 2016). This highlights the susceptibility of school students in higher class to smokeless tobacco use which could be related to their attitude in consuming smokeless tobacco. 

The findings of the present study revealed that higher proportion (16%) of females compared to (10.2%) of males consumed SLT although not statistically significant, which is in contrast to the findings of the cross-sectional study conducted by Ullah et al., (2018) in Bangladesh among 790 students of secondary schools aged 13- 18 years which reported ever SLT use as 10.2%in males and 8.3% in females. The higher proportion of SLT use observed in females could be due to the lesser sample of female participants in the study. Similar difference in the prevalence of ever tobacco use was found in other studies. Kumar et al., (2014) in Delhi reported the prevalence of SLT use to be 20.6% among males and 11.4 % among females. In another study conducted by Gupta (2018) based on Global Adult Tobacco Survey India (GATS-India) 2009-10 data among 15- 18 years old the prevalence of SLT use was found to be double among males (14%) as compared to females (7%) (Gupta, 2018).

The results of the present study revealed that a higher proportion of study subjects (13.7%) belonging to high socioeconomic class consumed SLT which is in accordance with the findings of the study conducted by Kumar et al., (2014) among 962 students of class 11th- 12th in schools of Delhi which also reported higher prevalence 13.6% of tobacco use among students of high socio-economic status.The increased use of tobacco with high socioeconomic status may be due to availability of money and higher spending capacity on tobacco among the study subjects .

The findings of the present study were in contrast to the study conducted by Daniel et al., (2008) in Kerala which reported that the prevalence of tobacco use was higher (24.2%) among those in the low socioeconomic group than in the middle (13.3%) and high socioeconomic groups (3.2%) (Daniel et al., 2008).

The results of this study revealed that exposure to tobacco (smoked/ smokeless) amongst peers, teachers and other school staff at school premise was significantly associated with the consumption of SLT in the study subjects which is in concordance with the results of study conducted by Hussain et al., (2017) among 2140 adolescents of class 6th- 10thand aged 11-16 years in schools of Karachi. The results of the study revealed that 33.6% of adolescents who used smokeless tobacco (SLT) and /or betel quid (BQ) saw their teacher using SLT and/or BQ in school.

It was observed that exposure to tobacco at home and outdoor public place was significantly associated with SLT use among study subjects which is in accordance with the findings of the previous conducted by Kowitt et al., (2019) which reported that adolescents who were exposed to secondhand smoke, compared to those who were not exposed, had greater odds of initiating SLT use (AOR: 2.71, 95% CI: 1.60, 4.59).

In this study, it was observed that there was significantly higher consumption of SLT among study subjects who were exposed through social media to tobacco advertisement/ promotion than other modes of media. It was different as compared to findings in the study conducted by Singh et al., (2015) which reported that television 58% was the main source of exposure for tobacco products followed by newspapers 26% and movies 16% . This difference in the results can be explained due to different study timings.

In conclusion, the total prevalence of smokeless tobacco use among study subjects was 11%. Consumption of smokeless tobacco was higher (16%) among females than males (10.2%) although not statistically significant. Factors such as exposure to tobacco at home, public places, at school and school teacher using smokeless tobacco (SLT), being exposed to tobacco advertisement and promotion via different mode of media was found to be associated with the use of SLT in the study subjects.

Thus, there is a dire need to intensify the activities for identification of current SLT users among school going adolescents in the community so as to identify the vulnerable groups, counsel them and catch them young for prevention and control of tobacco. School-based health promotion programme should be undertaken intensively to address and alert the vulnerable student population about the harmful effects of tobacco use. Lifestyle skills including refusal of tobacco products through skill-based programs for youth must be developed to empower them with ability to refrain from tobacco use.

## Author Contribution Statement

Conceptualization: AK,CD,PS; Design: AK,SG,CD,PS; Definition of intellectual content: AS,SG,MMS,CD; Literature search: AS,CD; Data acquisition: AS,CD; Data analysis: AS,SG,MMS,CD; Statistical analysis: AS,SG,MMS,CD; Manuscript preparation: AS,CD; Manuscript editing: AS,SG,MMS,CD; Manuscript review: SG,MMS,CD,PS,AB.
